# Days of Antibiotic Spectrum Coverage (DASC) and Oral Antimicrobial-Use Trends at a Community Pharmacy in Japan: A 2018–2023 Retrospective Observational Study

**DOI:** 10.3390/antibiotics14101051

**Published:** 2025-10-21

**Authors:** Kosuke Hasegawa, Shoji Seyama, Tomoko Mori, Yuriko Matsumura, Hidemasa Nakaminami

**Affiliations:** 1 MEDIX, Inc. 1-2-3 Motoyokoyamacho, Hachioji 192-0065, Tokyo, Japan; hasegawa.k@mdx-gr.com (K.H.); mori.t@mdx-gr.com (T.M.); matsumura.y@mdx-gr.com (Y.M.); 2 Department of Clinical Microbiology, School of Pharmacy, Tokyo University of Pharmacy and Life Sciences, 1432-1 Horinouchi, Hachioji 192-0392, Tokyo, Japan; sseyama@toyaku.ac.jp

**Keywords:** antimicrobial resistance, community pharmacy, antimicrobial stewardship, ASC score, DASC

## Abstract

Objective: The high frequency of prescribing oral antimicrobial agents, such as third-generation cephalosporins, macrolides, and fluoroquinolones, in local clinics is a major issue that can lead to the emergence of antimicrobial-resistant bacteria in the community. Hospitals have recently adopted the days of antibiotic spectrum coverage (DASC), which considers the antibacterial spectrum as a novel indicator of appropriate antimicrobial use. Although it has been used in inpatient settings, its applicability in community pharmacy settings remains unclear. Materials and Methods: The aim of this study was to determine whether the DASC is a valuable indicator of appropriate antimicrobial use in community pharmacies. We tabulated the use of antimicrobials dispensed at one of our pharmacies in Tokyo from 1 January 2018, to 31 December 2023. The DASC/100 prescriptions were calculated using the Antibiotic Spectrum Coverage score, which quantifies the extent of antimicrobial activity against key organisms. Higher scores indicate broader-spectrum agents, whereas lower scores indicate narrower-spectrum agents. Additionally, the days of therapy (DOT) value was calculated, along with the DOT/100 prescriptions, based on the dispensed prescriptions. Subgroup analyses were conducted for pediatric children aged < 6 years and the elderly (≥65 years). Results: The DASC/DOT was used to assess the appropriate use of antimicrobials. The DOT/100 and DASC/100 prescriptions in 2021–2023 were 50.1% and 51.5% lower, respectively, than those before 2020 (*p* < 0.05). During the same period, the DASC/DOT decreased by 0.7%, indicating that, despite the decrease in the number of antimicrobial prescriptions, the antimicrobial spectrum did not become narrower. In children < 6 years of age, DOT and DASC values declined significantly, possibly indicating a decrease in the number of antibiotic prescriptions for viral upper respiratory tract infections. In the elderly (≥65 years), the DASC/DOT remained relatively high, indicating their continued reliance on broad-spectrum agents, such as fluoroquinolones and macrolides. Conclusions: This study demonstrates the feasibility and benefits of the DASC/DOT as a spectrum-based indicator for appropriate antimicrobial use in community pharmacies. Therefore, the DASC/DOT serves as a practical and spectrum-sensitive indicator of outpatient antimicrobial use to guide antimicrobial stewardship in community settings. Furthermore, age-specific analyses highlighted the importance of targeted interventions to promote the judicious use of broad-spectrum antimicrobials, particularly among the elderly (≥65 years old).

## 1. Introduction

The emergence of antimicrobial-resistant bacteria is a significant global health concern that has prompted the implementation of antimicrobial resistance (AMR) countermeasures worldwide. In Japan, the AMR Control Action Plan was launched in 2016, and new outcome indicators were formulated in 2023 to achieve the objectives of the plan by 2027 [1,2]. In Japan, approximately 90% of antimicrobial prescriptions are issued in outpatient settings, and oral antimicrobials are prescribed more frequently in clinics than in hospitals [1,3]. Recent studies have further highlighted that clinical characteristics, such as the patient volume and specialty, influence antibiotic prescription patterns in primary care [4,5]. Third-generation cephalosporins, fluoroquinolones, and macrolides constituted 68.1% of all oral antimicrobial agents in 2023. Moreover, the use of oral antimicrobials declined from 2020 to 2022. However, prescriptions rebounded in 2023, with cephalosporin, fluoroquinolone, and macrolide use increasing by 14.7%, 25%, and 17.7%, respectively [6,7]. Furthermore, outpatient antimicrobial use in Japan remains higher than that in other countries [6]. Most of these prescriptions are for upper respiratory tract infections and diarrhea, which generally do not require antimicrobial therapy [3,8,9,10]. The spread of community-acquired methicillin-resistant *Staphylococcus aureus* (MRSA) in hospitals and geographic variation in antimicrobial prescriptions highlight the importance of community-specific surveillance [6,11,12]. Further, understanding local antimicrobial prescription patterns in community clinics and pharmacies is essential for promoting appropriate antimicrobial use [13].

Several metrics are available to evaluate antimicrobial use in hospitals, such as the antimicrobial use density (AUD) and days of therapy (DOT) [14,15,16]. However, until recently, standardized indices were lacking in community settings. Then, defined daily doses (DDDs)/100 prescriptions per month (DPM) and DDDs/100 prescriptions per year (DPY) were introduced [12,17]. However, these volume-based indices require calculations based on drug potency and do not consider the antimicrobial spectrum. Consequently, they might not adequately assess the appropriateness of antimicrobial use, especially when evaluating narrowing of the antimicrobial spectrum from broad-spectrum to narrow-spectrum agents.

The days of antibiotic spectrum coverage (DASC) is a novel indicator for antimicrobial stewardship teams [18]. Unlike conventional indices, the DASC incorporates the antimicrobial spectrum of each agent, enabling a qualitative assessment of prescription patterns. The Antibiotic Spectrum Coverage (ASC) score underlying the DASC reflects its activity against clinically important organisms, including *S. aureus*, *Streptococcus pneumoniae*, *Escherichia coli*, and MRSA, which are relevant in both hospital and community settings. Higher ASC scores indicate a broader spectrum of activity, whereas lower scores correspond to a narrower spectrum. Integrating the ASC score into the DASC has provided a spectrum-adjusted measurement of antimicrobial use. DASC/DOT represents the average antimicrobial spectrum per prescription. A higher DASC/DOT value indicates the preferential use of broad-spectrum agents, whereas a lower value indicates the predominant use of narrow-spectrum agents. Thus, the DASC/DOT is a clinically interpretable measure of prescription quality that complements volume-based indices. Previous studies demonstrated the utility of the DASC for inpatient care. However, their application in community pharmacies remains unexplored. Moreover, the relationship between the DOT and DASC has not yet been examined to evaluate the appropriateness of antimicrobial use in outpatient settings [18,19,20,21,22,23]. The DASC/DOT may enable community pharmacists to identify targets for improving antimicrobial selection, even in outpatient settings where de-escalation strategies are rarely employed. Thus, the objective of this study was to evaluate the usefulness of the DASC/DOT in community pharmacies and to determine whether it can effectively capture changes in the antimicrobial spectrum that conventional volume-based indicators cannot detect.

## 2. Methods

### 2.1. Patients and Study Period

The study protocol was approved by the Ethics Committee of the Pharmacy Society of Japan (24005) on 4 March 2024. For the purposes of this study, a “prescription” refers to a single dispensing event at a community pharmacy. This event may include a full course of antimicrobial therapy regardless of the number of days of treatment prescribed by the physician. Prescription details were tabulated using only the date on which the antibiotic was dispensed, age, sex, and drug name to protect the identities of the individuals. No personally identifiable information (such as drug history) was provided.

This retrospective observational study was performed to analyze the dispensing data of a single community pharmacy located near a clinic providing community-based medical care in the Tama area of Tokyo from 2018 to 2023. The clinical departments encompass various specialties including internal medicine, cardiovascular medicine, respiratory medicine, and pediatrics. The clinic has been an outpatient fever clinic since the onset of the coronavirus disease-2019 (COVID-19) pandemic. The dataset, spanning January 2018 to December 2023, included information regarding the dispensing date, patient age and sex, antimicrobial agent name, tablet count, day supply, and total number of prescriptions dispensed per month. Importantly, this study was limited to an analysis of oral antimicrobials and excluded injections, topical antimicrobials, antifungals, and antiparasitic agents.

### 2.2. Antimicrobial Use Metric Calculation

The DASC score was calculated using the previously defined ASC score [16,18,19,20,24]. The calculation yielded the DASC/100 and DOT/100 prescriptions, representing the total antimicrobial spectrum and duration of administration per 100 prescriptions, respectively. Annual trends in the average antimicrobial spectrum per prescription, represented by the DASC/DOT, were analyzed to assess any narrowing of any spectrum over time. Previously proposed DPM and DPY indicators of antimicrobial use in community pharmacies were also calculated [12,17]. Specifically, the DPM was calculated as the total DDDs of each antimicrobial dispensed in a given month divided by the total number of prescriptions in that month and multiplied by 100. The DPY was calculated by dividing the total DDDs of each antimicrobial dispensed in a given year by the total number of prescriptions in that year and multiplying by 100. For the study population, all prescriptions dispensed at community pharmacies during the study period (2018–2023) were included. Analyses were stratified by age group to capture age-specific antimicrobial use patterns, including children aged < 6 years and the elderly (≥65 years).

### 2.3. Statistical Analyses

All statistical analyses were performed using Microsoft Excel (Microsoft Corporation, Redmond, WA, USA). The Mann–Whitney U test was used to evaluate the differences in the DASC/DOT and other antimicrobial use indicators between individual years and the two periods (2018–2020 and 2021–2023). Each monthly value was treated as an independent data point. This nonparametric test was selected because the data did not follow a normal distribution. Statistical significance was set at *p* < 0.05. Linear regression analysis was used to assess the temporal trends (2018–2023) to determine the antimicrobial use changes over time for specific age groups (children aged < 6 years and the elderly aged ≥65 years). The trend significance was determined using the *p*-value of the regression slope. Correlations between the DASC/DOT and other indicators of antimicrobial use (DPM, DPY, and DOT/100 prescriptions) were also analyzed. Additionally, Spearman’s rank correlation coefficient (*ρ*) was used to evaluate the monotonic relationships between antimicrobial use indicators. A correlation coefficient of −0.2 ≤ *ρ* ≤ 0.2 (inclusive) was considered insignificant, indicating no correlation.

## 3. Results

### 3.1. Annual Indicator Changes

The number of non-antimicrobial prescriptions remained relatively consistent during the study period, as indicated by the recorded figures (2018: 23,195; 2019: 25,884; 2020: 21,261; 2021: 22,491; 2022: 25,956; 2023: 27,999). Table 1 presents data on the number of prescriptions received and antimicrobials provided at a pharmacy. Although no statistically significant change was observed in the median number of prescriptions received between 2018 and 2023, an increasing trend was observed in 2022 (2179 [2065.3–2281]) and 2023 (2326 [2185.5–2440.3]). Conversely, the median number of antimicrobial prescriptions decreased substantially from 327 (306.5–346.3) in 2019 to 139.5 (128.3–173.5) in 2020. The median DOT/100 prescriptions also showed a downward trend, declining from 91.6 (81.6–105.2) in 2018 to 28.4 (25.4–29.9) in 2022, before increasing to 38.8 (32.4–43.2) in 2023. Similarly, the median DASC/100 prescriptions declined annually from 657.9 (567.1–720.9) in 2018 to 202.3 (191.2–222.6) in 2022 and subsequently increased to 265.6 (231.2–326) in 2023. The median DASC/DOT increased from 7.0 (6.8–7.2) in 2018 to 7.3 (7.0–7.5) in 2022. Meanwhile, the median DPY declined from 74.2 (66.7–86.2) in 2018 to 24.2 (22.2–25.5) in 2022, subsequently increasing to 32.3 (29.2–38.9) in 2023.

**Figure 1:**
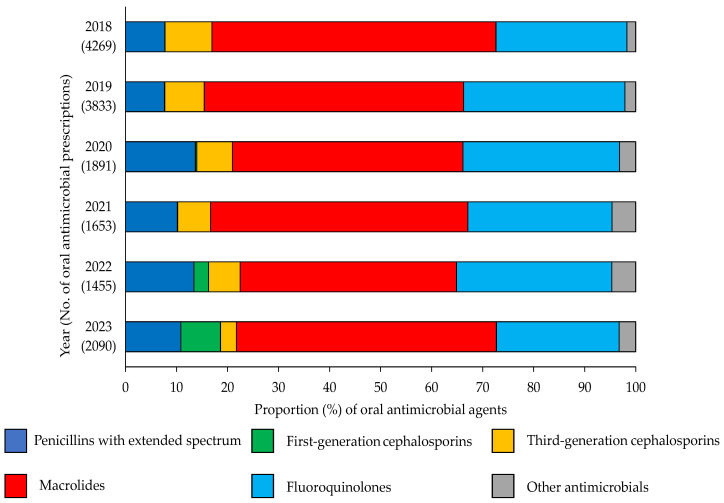
Proportions of different oral antimicrobial agents prescribed between 2018 and 2023 at a pharmacy.

Figure 1 shows the prescription distribution based on the antimicrobial class. “Other antimicrobials” includes antibiotics with relatively low prescription volumes, such as second-generation cephalosporins, combinations of penicillins (including β-lactamase inhibitors), other cephalosporins and penems, fosfomycin, tetracyclines, and combinations of sulfonamides and trimethoprim, including their derivatives. The number of third-generation cephalosporin, macrolide, and fluoroquinolone prescriptions decreased significantly (*p* < 0.05) between 2018–2020 and 2021–2023, with reductions of 68.1% (822–262), 51.4% (5179–2515), and 51.1% (2888–1413), respectively. Conversely, the number of first-generation cephalosporin prescriptions increased significantly (*p* < 0.05) from seven (0.1%) to 205 (3.9%) between the same periods.

The number of prescriptions for third-generation cephalosporins, macrolides, and fluoroquinolones decreased markedly, while the number of prescriptions for first-generation cephalosporins increased. “Other antimicrobials” refers to agents with relatively low prescription volumes.

### 3.2. Overall Trends of Antibiotic Use in a Pharmacy

**Figure 2:**
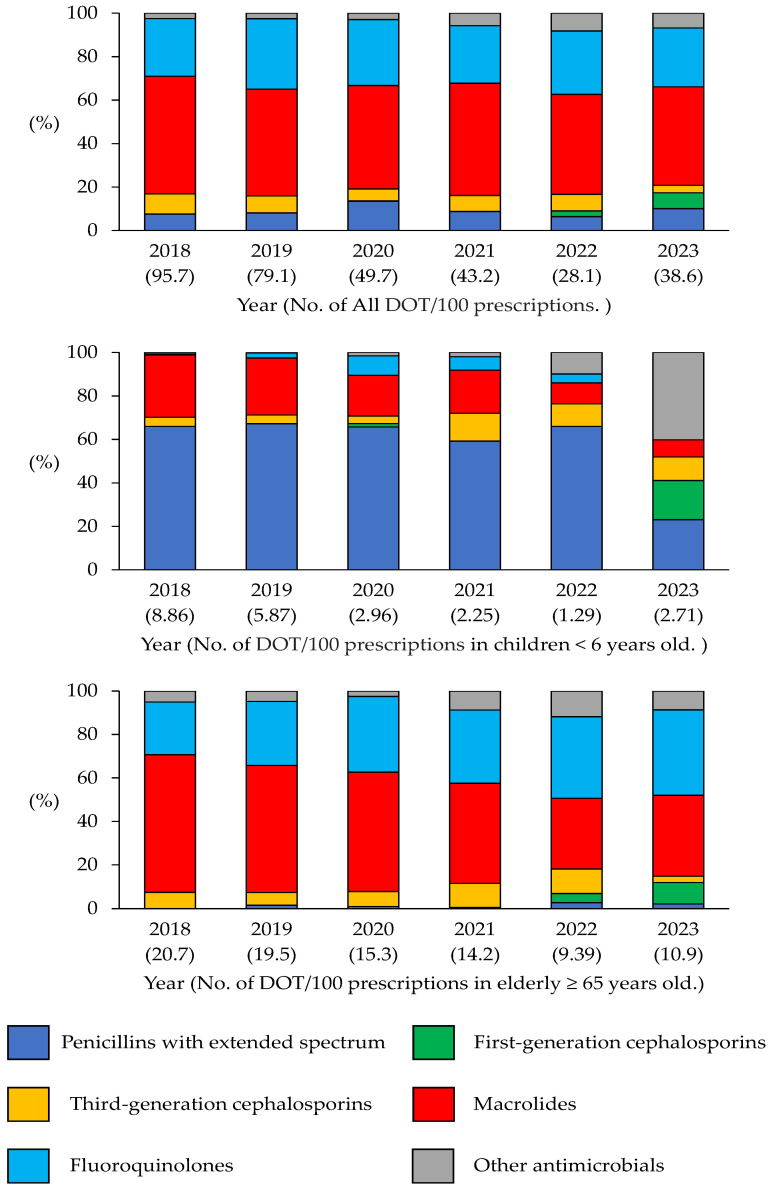
Days of therapy (DOT)/100 prescriptions between 2018 and 2023.

The DOT/100 prescriptions was calculated based on the dispensed antimicrobial prescriptions (Figure 2). A comparison of the overall DOT/100 prescriptions revealed a significant decrease of 51.1% (*p* < 0.05) between 2018–2020 and 2021–2023. Subsequently, a comparative analysis of the DOT/100 prescriptions in 2018–2020 and 2021–2023 was conducted for each antibacterial class. The results revealed a notable decline in the DOT/100 prescriptions for third-generation cephalosporins, fluoroquinolones, and macrolides, with reductions of 62.8%, 54.6%, and 53.7%, respectively (*p* < 0.05). Conversely, the DOT/100 prescriptions for first-generation cephalosporins increased markedly from 0.17 (0.08%) to 3.59 (3.27% (*p* < 0.05) in the same periods.

Trends in DOT/100 prescriptions from 2018 to 2023 are presented for all patients, as well as for children (under 6 years old) and older adults (65 years and older). Overall, as well as for each class, the DOT/100 prescription values decreased, especially for broad-spectrum antibiotics.

**Figure 3:**
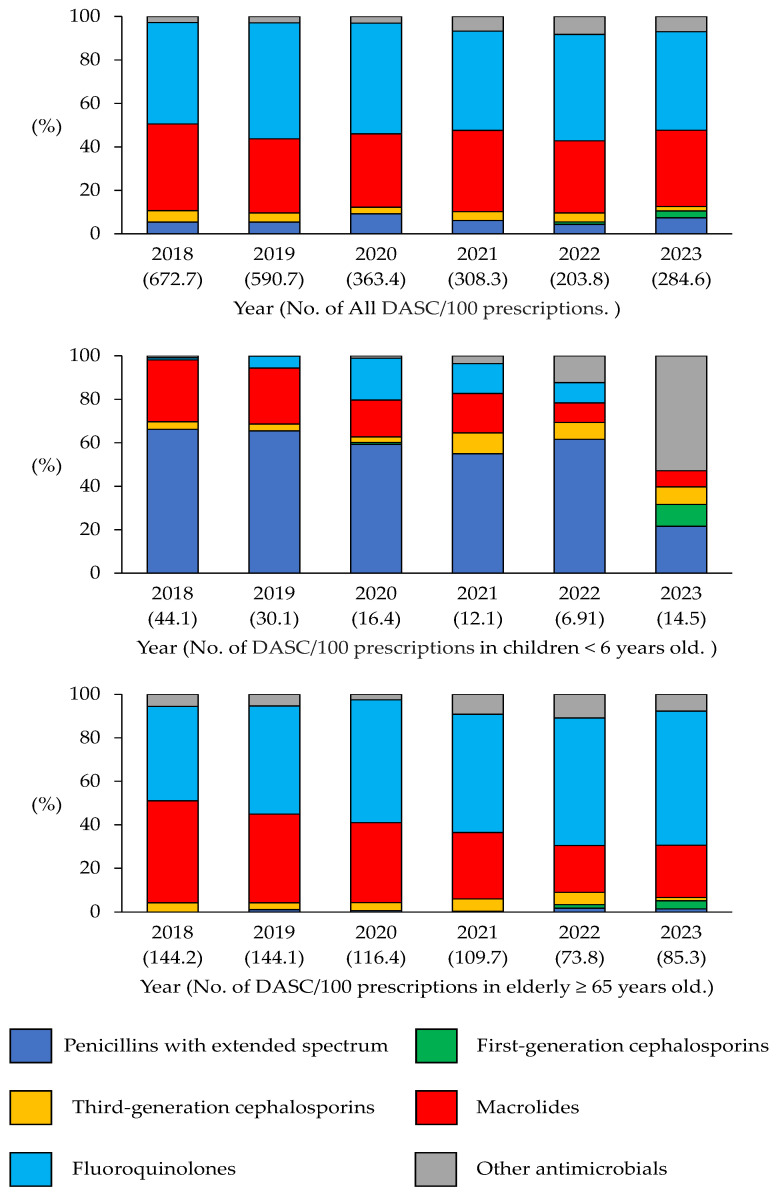
Days of antibiotic spectrum coverage (DASC)/100 prescriptions between 2018 and 2023.

An analysis of all antimicrobial prescriptions in the DASC/100 prescription database during the same period revealed a notable decline of 51.5% in 2021–2023 (*p* < 0.05; Figure 3). The same classification, as in Figure 1, was used, and a comparative analysis of the DASC/100 prescriptions in 2018–2020 and 2021–2023 was conducted for each antibacterial class. The results revealed a significant decrease in the number of prescriptions of third-generation cephalosporins, fluoroquinolones, and macrolides, with reductions of 62.2%, 54.1%, and 52.2%, respectively (*p* < 0.05). In contrast, the DASC/100 prescriptions for first-generation cephalosporins increased considerably from 0.52 (0.03%) to 11.4 (1.45%; *p* < 0.05) between the same periods.

The number of DASC/100 prescriptions decreased significantly across most classes, reflecting the use of a narrower antimicrobial spectrum. After 2021, a modest increase in first-generation cephalosporins was observed.

**Figure 4:**
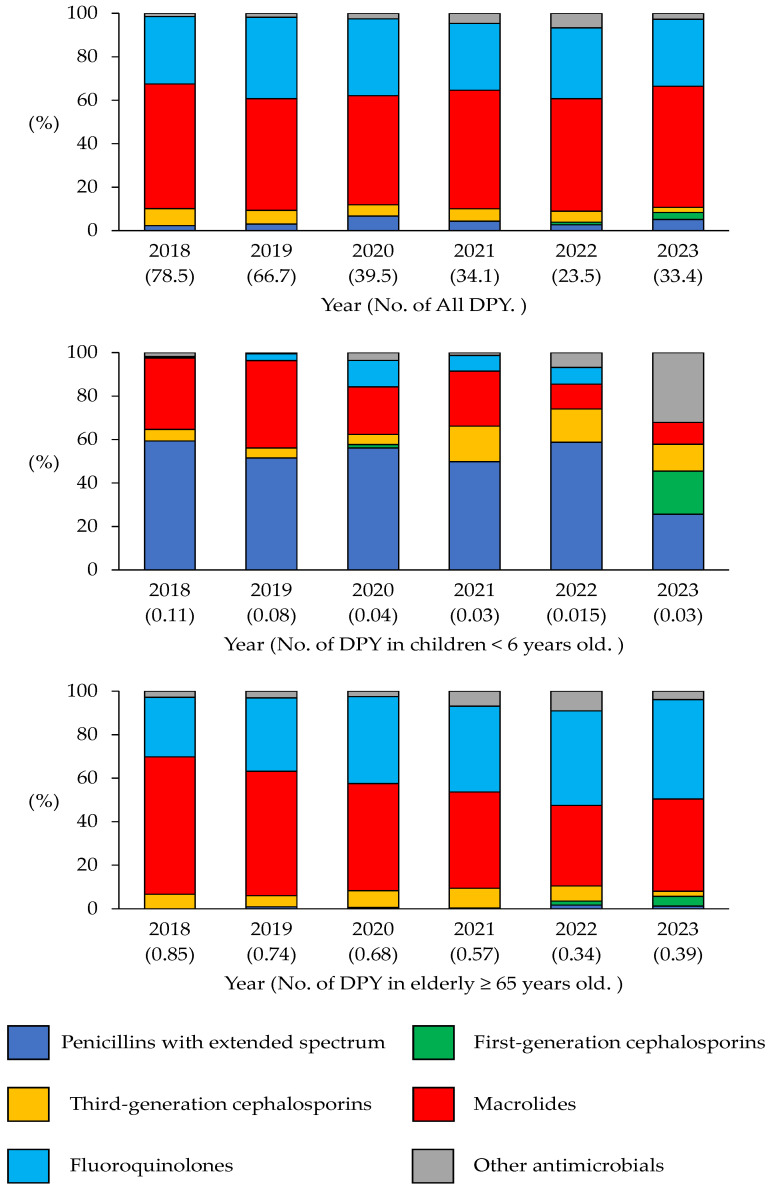
Defined daily doses (DDDs)/100 prescriptions/year (DPY) between 2018 and 2023.

Figure 4 illustrates the annual trends in the DPY. The same classification shown in Figure 1 was used. A comparison of the total DPY revealed a significant decrease of 50.8% (*p* < 0.05), between the 2018–2020 and 2021–2023 periods. Between the same periods, when the DPY was evaluated by antimicrobial class, significant reductions of 68.3%, 50.2%, and 55.2% were observed in the DPY for third-generation cephalosporins, fluoroquinolones, and macrolides, respectively (*p* < 0.05). Moreover, the DPY for first-generation cephalosporins notably increased from 0.08 (0.04%) to 11.4 (1.51%; *p* < 0.05) between the same periods.

Over time, DPY declined in all age groups, primarily due to the decreased use of broad-spectrum agents. However, there has been a slight increase in the use of first-generation cephalosporins in recent years.

### 3.3. Trends of Antibiotic Use in Children Aged < 6 Years

Figure 2 illustrates the annual trends in DOT/100 prescriptions for children aged < 6 years from 2018 to 2023. The total DOT/100 prescriptions decreased from 8.86 in 2018 to 2.71 in 2023, indicating a 69.4% reduction over the 6-year period (*p* < 0.05). Among the individual antimicrobial classes, extended-spectrum penicillin DOT/100 prescriptions showed a marked reduction from 5.85 in 2018 to 0.63 in 2023, indicating an 89.2% reduction (*p* < 0.05). Similarly, the DOT/100 prescriptions for macrolides declined substantially from 2.54 in 2018 to 0.21 in 2023, reflecting a 91.7% reduction (*p* < 0.05). In contrast, the DOT/100 prescriptions for third-generation cephalosporins showed a relatively stable trend, slightly decreasing from 0.38 in 2018 to 0.29 in 2023. However, this change was not statistically significant (*p* = 0.23).

Figure 3 shows the annual trend in the DASC/100 prescriptions for children aged < 6 years from 2018 to 2023. The total DASC/100 prescriptions decreased from 44.16 in 2018 to 14.53 in 2023, indicating a significant reduction of 67.1% over the 6-year period (*p* < 0.05). Among the individual antimicrobial classes, the DASC/100 prescriptions for extended-spectrum penicillins showed a substantial decline from 29.23 in 2018 to 3.13 in 2023, indicating an 89.3% reduction (*p* < 0.05). Similarly, the DASC/100 prescriptions for macrolides was markedly reduced from 12.58 in 2018 to 1.07 in 2023, indicating a 91.5% reduction (*p* < 0.05). In contrast, the DASC/100 prescriptions for third-generation cephalosporins remained relatively stable, decreasing only slightly from 1.52 in 2018 to 1.17 in 2023; however, this change was not statistically significant (*p* = 0.28).

Figure 4 shows the annual trend in the DPY in children aged < 6 years from 2018 to 2023. The total DPY decreased from 0.113 in 2018 to 0.026 in 2023, representing a significant reduction of approximately 76.8% (*p* < 0.05). The DPY for extended-spectrum penicillins decreased from 0.067 in 2018 to 0.0067 in 2023, indicating a substantial decrease of approximately 90.0% (*p* < 0.05). The DPY for macrolide antibiotics also decreased from 0.037 in 2018 to 0.0026 in 2023, a significant reduction of approximately 92.9% (*p* < 0.05). Although the DPY of third-generation cephalosporins decreased from 0.006 in 2018 to 0.0032 in 2023, this change was not statistically significant (*p* = 0.28). The results of the linear regression analysis of the DOT/100 prescriptions, DASC/100 prescriptions, and DPY for children aged < 6 years are summarized in Table 2.

### 3.4. Trends of Antibiotic Use in the Elderly (≥65 Years)

Figure 2 shows the annual trends in systemic antibiotic DOT/100 prescriptions in the elderly (≥65 years) from 2018 to 2023. The total DOT/100 prescriptions decreased from 20.71 in 2018 to 10.91 in 2023, marking an approximate 47% reduction over the 6-year period (*p* < 0.05). Although a consistent downward trend was observed from 2018 to 2022, the DOT/100 prescriptions slightly increased in 2023. Macrolides were associated with the highest DOT/100 prescriptions, which markedly decreased from 13.10 in 2018 to 4.06 in 2023, indicating a 69.0% reduction (*p* < 0.05). The DOT/100 prescriptions for fluoroquinolones also decreased from 5.02 in 2018 to 4.29 in 2023, marking a modest 14.6% reduction (*p* = 0.08). The DOT/100 prescriptions for third-generation cephalosporins decreased significantly from 1.54 in 2018 to 0.33 in 2023, indicating a 78.6% reduction (*p* < 0.05). In contrast, the DOT/100 prescriptions of first-generation cephalosporins, which were rarely used until 2021, showed a notable increase from 0.39 in 2022 to 1.05 in 2023 (*p* < 0.05).

Figure 3 shows the annual trend in the DASC/100 prescriptions for systemic antimicrobial agents in the elderly (≥65 years) from 2018 to 2023. The total DASC/100 prescriptions decreased from 144.17 in 2018 to 85.33 in 2023, representing an approximate 40.8% reduction over the 6-year period (*p* < 0.05). Although a consistent downward trend was observed until 2022, the DASC/100 prescriptions slightly increased in 2023. In terms of the antibiotic class, macrolides exhibited the highest DASC/100 prescriptions in 2018 of 67.35, which decreased to 20.43 in 2023, marking a 69.7% reduction (*p* < 0.05). The DASC/100 prescriptions for fluoroquinolones also declined from 62.64 in 2018 to 52.62 in 2023, indicating a 16.0% decrease (*p* = 0.07). The DASC/100 prescriptions of third-generation cephalosporins showed a marked decrease from 6.16 in 2018 to 1.30 in 2023, indicating a 78.9% reduction (*p* < 0.05). In contrast, the DASC/100 prescriptions of first-generation cephalosporins, which were rarely prescribed until 2021, increased from 1.18 in 2022 to 3.16 in 2023 (*p* < 0.05).

Figure 4 shows the annual trends in the DPY for systemic antimicrobial agents in the elderly(≥65 years of age) from 2018 to 2023. The total DPY decreased from 0.854 in 2018 to 0.388 in 2023, and linear regression analysis revealed a significant decrease of 54.6% (*p* < 0.05). Macrolide antibiotics were associated with a significant decrease in the DPY of 69.5%, from 0.539 in 2018 to 0.164 in 2023 (*p* < 0.05). The DPY for fluoroquinolone antibiotics also decreased from 0.233 in 2018 to 0.178 in 2023, indicating a 23.8% reduction, but this was not statistically significant (*p* = 0.09). Similarly, the DPY for third-generation cephalosporins decreased significantly from 0.057 in 2018 to 0.009 in 2023 (*p* < 0.05). In contrast, first-generation cephalosporins were rarely used until 2021, but their DPY significantly increased from 0.0063 in 2022 to 0.02 in 2023 (*p* < 0.05). The results of the linear regression analysis for the DOT/100 prescriptions, DASC/100 prescriptions, and DPY in older adults aged ≥ 65 years are summarized in Table 3.

### 3.5. Annual Trends in the DASC/DOT and Correlations

**Figure 5:**
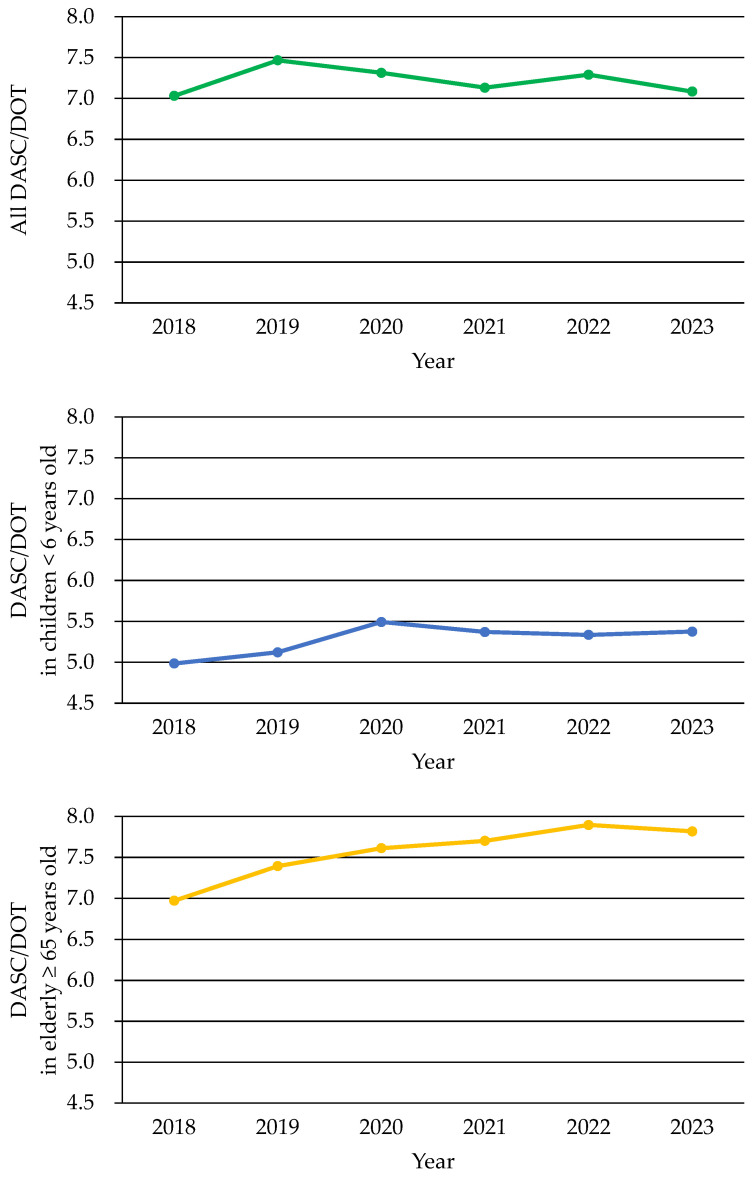
Days of antibiotic spectrum coverage (DASC)/days of therapy (DOT) between 2018 and 2023.

Figure 5 illustrates the annual changes in the DASC/DOT over 6 years, representing the average antimicrobial spectrum per prescription. A comparison between the 2018–2020 and 2021–2023 periods revealed a 0.7% decrease in the overall DASC/DOT, which was not statistically significant (*p* = 0.67). When stratified by age, children under 6 years of age showed an increase in the DASC/DOT from 2018 to 2020, followed by no notable change from 2021 to 2023. Over the entire period, the DASC/DOT increased by 3.08% in this age group; however, this change was not statistically significant (*p* = 0.35). In contrast, individuals aged ≥ 65 years demonstrated a consistent annual increase in the DASC/DOT, with a 12.2% increase observed between 2018 and 2023 (*p* = 0.07). These findings suggest age-related differences in antimicrobial spectrum trends, with the DASC/DOT remaining relatively stable in young children but increasing in the elderly (≥65 years).

Green indicates total DASC/DOT, blue indicates children <6 years old, and yellow indicates elderly ≥65 years old. The overall DASC/DOT remained stable, though it gradually increased among older adults. This suggests that there are age-related differences in antimicrobial spectrum patterns.

Table 4 shows the correlation between the DASC/DOT and each indicator. The correlation coefficient between the DOT/100 prescriptions and DASC/DOT was *ρ* = 0.05, indicating no significant correlation between the total number of prescriptions and number of antimicrobial prescription days (*p* = 0.67). The correlation coefficient between the DPM (local antimicrobial use indicator) and DASC/DOT was *ρ* = 0.1, indicating no significant correlation between the decrease in the DDDs of prescribed antimicrobials and number of antimicrobial prescription days (*p* = 0.85). The correlation coefficient between the DPY and DASC/DOT was *ρ* = −0.12, revealing no significant correlation between the prescribed antimicrobial DDDs and number of antimicrobial prescription days (*p* = 0.82). The corresponding scatter plots for these correlations are shown in Figure S1 and Table 4.

## 4. Discussion

In this study, we evaluated antimicrobial prescription patterns and assessed the utility of spectrum- and volume-based indicators (e.g., DASC/DOT, DOT/100 prescriptions, and DPY) in community pharmacies between 2018 and 2023. Overall, third-generation cephalosporins, macrolides, and fluoroquinolones showed a marked decline in prescriptions after 2020, whereas first-generation cephalosporins increased. The “Other antimicrobials” category remained relatively stable, except for a notable increase among young children in 2023. These trends reflect both temporal changes in prescription patterns, including the effect of the COVID-19 pandemic, and the influence of pharmacist-led stewardship interventions.

The decline in the number of antimicrobial prescriptions in the assessed pharmacy in 2020 was likely because of restrictions imposed due to the COVID-19 pandemic (Table 1). Nevertheless, the annual number of dispensed prescriptions exceeded 20,000, and no significant difference was observed in the total prescription numbers between the pre- and post-COVID-19 periods (*p* = 0.39). This indicates that the decline in antimicrobial prescriptions was not simply due to a reduction in the number of prescriptions accepted by community pharmacies. The deployment of antigen test kits and screening protocols for patients with acute symptoms may have contributed to a reduction in unnecessary antimicrobial prescriptions in patients with COVID-19.

Notably, the numbers of antimicrobial, DOT/100, and DASC/100 prescriptions declined until 2022, followed by an increase in 2023. This trend was possibly due to increased human contact after COVID-19 reclassification as a Category 5 infection in Japan in May 2023, with a resurgence of bacterial infections that had previously declined during the pandemic (Table 1). Indeed, the antibiotic demand temporarily decreased due to the reduced prevalence of common infectious diseases, whereas the resurgence of previously uncommon infectious diseases led to sudden increases in the oral antibiotic demand, causing supply instability [13,25].

Although the overall volume of antimicrobial prescriptions decreased, the DASC/DOT did not decline, suggesting the preferential use of broad-spectrum antibiotics. Therefore, DASC/DOT monitoring in community pharmacies is useful for qualitatively assessing prescription practices. For example, the aggregate DOT/100 and DASC/100 prescriptions decreased during the study period; however, DASC/DOT remained unchanged, indicating that broad-spectrum antimicrobial use was consistently maintained.

From 2022 to 2023, the proportion of first-generation cephalosporin prescriptions increased significantly. This increase was likely influenced by the pharmacist’s recommendation of cephalexin use to prescribing physicians. This suggests that pharmacists can actively influence antimicrobial stewardship. Although nationwide or multicenter data are not currently available to confirm whether similar trends have occurred elsewhere, these findings highlight the tangible impact that a single community pharmacist can have on local antimicrobial prescribing practices. These initiatives are consistent with the goals of the National Action Plan on Antimicrobial Resistance (2023–2027) [1], which emphasizes community-level antimicrobial stewardship and close collaboration between physicians and pharmacists to promote the use of narrow-spectrum agents when appropriate. Therefore, this observation suggests that community pharmacists can play an active and influential role in promoting the appropriate use of antimicrobials in outpatient settings. Additionally, in 2023, a sudden increase in the “Other antimicrobials” category among children under 6 years of age was observed. This was primarily due to the prolonged prescription of sulfamethoxazole/trimethoprim to patients who had undergone transplant surgery, reflecting the specific needs of this population.

The Pediatric Antimicrobial Stewardship Support Add-on may have contributed to a reduction in the use of broad-spectrum antibiotics, such as tosufloxacin and tebipenem. Furthermore, since the options for broad-spectrum agents are limited in pediatric patients, the DASC/DOT values are suggested to be lower compared with those observed in the overall population and in the elderly (≥65 years).

Additionally, COVID-19 influenced the selection of specific antimicrobial classes. For example, fluoroquinolone prescriptions for pneumonia treatment and prevention increased early during the pandemic after positive antigen tests, and subsequent increases in levofloxacin and lascufloxacin prescriptions were associated with COVID-19 treatment regimens [26,27]. However, the precise causal effect of the COVID-19 reclassification as a Category 5 infectious disease on these prescription patterns requires further investigation. These findings emphasize the importance of promoting the appropriate use of antimicrobials, particularly in the elderly population.

One practical approach is stepwise escalation therapy, in which treatment is initiated with narrow-spectrum agents (e.g., extended-spectrum penicillins and first- or second-generation cephalosporins, which have low ASC scores) and escalated to broad-spectrum antibiotics only if the clinical response is inadequate. This strategy can reduce unnecessary broad-spectrum antibiotic use and mitigate the emergence of antimicrobial-resistant organisms in communities.

Previous hospital-based studies have reported DASC/DOT values of 7.0–8.5, suggesting that interventions to promote narrow-spectrum alternatives to fluoroquinolones and macrolides (high ASC scores, targets of the AMR Action Plan) may further reduce the DASC/DOT [14,18,20,21]. Moreover, some classes, such as macrolides, are often used long-term at low doses for chronic sinusitis in otolaryngology or nontuberculous mycobacterial infections in respiratory medicine [28,29]. This may partly explain why substantial changes in the DASC/DOT were not observed in specific patient groups. Future studies including multiple pharmacies and diverse patient populations are necessary to validate these findings.

Recently, the DPM and DPY were proposed as indicators of antimicrobial use in community pharmacies. Because these indices are based on prescription counts, we examined their relationship with the DASC/DOT. Consistent with results based on prior inpatient studies, no significant correlations were observed between the DASC/DOT and DPM or DPY [18]. This suggests that the DASC/DOT maintains distinct measurement characteristics across settings, thereby supporting its external validity. Unlike the DPM and DPY, which only assess quantity, the DASC/DOT integrates both the number of prescription days and the antimicrobial spectrum, offering a qualitative perspective [12,17]. Therefore, a combined evaluation of spectrum- and volume-based metrics can provide a more comprehensive understanding of antimicrobial use.

This study had some limitations. First, as the first investigation of the DASC in a community pharmacy setting, direct comparisons with other studies are limited. Second, as a single-site study, further research based on multiple pharmacies is required. Third, clinical information, such as the diagnosis, severity, and culture results, was unavailable, making it difficult to assess the appropriateness of each prescription. Despite these limitations, our findings demonstrate that the DASC/DOT can serve as a robust indicator of antimicrobial use in community pharmacies, complementing volume-based indices such as the DPM and DPY. To further clarify the practical value of each indicator, we summarize their characteristics, strengths, limitations, and usefulness in community pharmacy settings in Table 5.

This tabular presentation enables the readers to compare and validate the applicability of each metric as a surveillance tool. The integration of these indicators into pharmacy practices could support pharmacists in promoting optimal antimicrobial use and contribute to AMR countermeasures at the community level.

## Figures and Tables

**Table 1:** Distributions (median and interquartile range) of antimicrobial consumption in a community pharmacy.

Characteristic or Metric	Median (IQR: Interquartile Range)					
	2018	2019	2020	2021	2022	2023
Number of total prescriptions received (monthly median, IQR)	1894 (1747.3–2077.8)	2191 (1951–2254)	1739 (1672–1887.8)	1884.5 (1801.5–1972.8)	2179 (2065.3–2281)	2326 (2185.5–2440.3)
Number of antimicrobial prescriptions received (monthly median, IQR)	320 (291–391.3)	327 (306.5–346.3)	139.5 (128.3–173.5)	144 (133–152.3)	109 (99–121.3)	172.5 (157–194)
DOT/100 prescriptions (median, IQR)	91.6 (81.6–105.2)	83.1 (73.6–85.6)	46.2 (43.1–52.2)	43.5 (41.4–45.7)	28.4 (25.4–29.9)	38.8 (32.4–43.2)
DASC/100 prescriptions (median, IQR)	657.9 (567.1–720.9)	614.3 (555.6–647)	330.4 (304.4–376.3)	310.2 (286.1–330.2)	202.3 (191.2–222.6)	265.6 (231.2–326)
DASC/DOT (median, IQR)	7.0 (6.8–7.2)	7.4 (7.8–7.5)	7.2 (7.0–7.4)	7.1 (6.8–7.3)	7.3 (7.0–7.5)	7.2 (7.0–7.4)
DPY (median, IQR; DDDs/100 prescriptions/year)	74.2 (66.7–86.2)	69.4 (62.8–71.6)	35.0 (33.9–42.4)	33.7 (32.6–36.0)	24.2 (22.2–25.5)	32.3 (29.2–38.9)

Abbreviations: DOT, days of therapy; DASC, days of antibiotic spectrum coverage; DDDs, defined daily dose; DPY, DDDs/100 prescriptions/year. Values are presented as the median (interquartile range, IQR), calculated from the monthly totals of prescriptions at the community pharmacy. The DOT/100 and DASC/100 prescriptions are based on all oral antimicrobial prescriptions. The DPY represents the defined daily dose (DDD) per 100 prescriptions per year.

**Table 2:** Linear regression results of antimicrobial use indicators in children < 6 years of age (2018–2023).

Indicator/Antibiotic Class	Slope	R^2^	*p*-Value
DOT/100 prescriptions			
Total	−1.07	0.92	<0.05
Extended-spectrum penicillin	−0.89	0.94	<0.05
Third-generation cephalosporins	−0.02	0.29	0.23
Macrolides	–0.41	0.89	<0.05
DASC/100 prescriptions			
Total	−5.01	0.90	<0.05
Extended-spectrum penicillin	−5.36	0.93	<0.05
Third-generation cephalosporins	−0.07	0.35	0.28
Macrolides	−2.03	0.91	<0.05
DPY			
Total	−0.01	0.90	<0.05
Extended-spectrum penicillin	−0.01	0.93	<0.05
Third-generation cephalosporins	−0.001	0.35	0.28
Macrolides	−0.004	0.91	<0.05

Abbreviations: DOT, days of therapy; DASC, days of antibiotic spectrum coverage; DPY, defined daily doses/100 prescriptions/year.

**Table 3:** Linear regression results of antimicrobial use indicators in the elderly (≥65 years; 2018–2023).

Indicator/Antibiotic Class	Slope	R^2^	*p*-Value
DOT/100 prescriptions			
Total	−1.62	0.91	<0.05
First-generation cephalosporins	+0.13	0.67	<0.05
Third-generation cephalosporins	−0.22	0.71	<0.05
Macrolides	−1.55	0.92	<0.05
Fluoroquinolones	−0.13	0.42	0.08
DASC/100 prescriptions			
Total	−9.98	0.88	<0.05
First-generation cephalosporins	+0.38	0.63	<0.05
Third-generation cephalosporins	−0.98	0.75	<0.05
Macrolides	−7.94	0.90	<0.05
Fluoroquinolones	−2.11	0.56	0.07
DPY			
Total	−0.11	0.93	<0.05
First-generation cephalosporins	+0.003	0.64	<0.05
Third-generation cephalosporins	−0.01	0.63	<0.05
Macrolides	−0.08	0.93	<0.05
Fluoroquinolones	−0.02	0.53	0.09

Abbreviations: DOT, days of therapy; DASC, days of antibiotic spectrum coverage; DPY, defined daily doses/100 prescriptions/year.

**Table 4:** Correlations between DASC/DOT and each indicator.

	Correlation Coefficient (*ρ*)	*p*-Value
DOT/100 prescriptions vs. DASC/DOT	0.05	0.67
DPM vs. DASC/DOT	0.1	0.85
DPY vs. DASC/DOT	−0.12	0.82

Abbreviations: DASC, days of antibiotic spectrum coverage; DOT, days of therapy; DPM, defined daily doses (DDDs)/100 prescriptions/month; DPY, DDDs/100 prescriptions/year.

**Table 5:** Characteristics, strengths, limitations, and usefulness of antibiotic surveillance metrics in community pharmacies.

Metric	Feature	Strengths	Limitations	Usefulness in Community Pharmacies
DOT	Measures days of therapy	Easy to calculate, standard in hospitals	Does not reflect antimicrobial spectrum	Useful for quantifying overall antimicrobial use
DASC	Considers the antimicrobial spectrum	Enables spectrum-based evaluation, qualitative assessment	Limited evidence, especially in community settings; further validation is needed	Useful for evaluating the quality of antimicrobial use
DPM	DDDs/100 prescriptions/month	Enables monthly comparisons	Based on DDD, which may lead to discrepancies depending on the actual prescribed amount or duration; Affected by seasonal variation	Useful for monitoring short-term trends
DPY	DDDs/100 prescriptions/year	Enables annual comparisons, less affected by seasonality	Based on DDD, which may lead to discrepancies depending on the actual prescribed amount or duration	Useful for long-term evaluation and less affected by seasonal variation

DASC, days of antibiotic coverage; DOT, days of therapy; DDD, defined daily dose; DPM, DDDs/1000 prescriptions/month; DPY, DDDs/1000 prescriptions/year.

## Data Availability

The original contributions presented in this study are included in the article.

## Supplementary Materials

The following supporting information can be downloaded at: https://www.mdpi.com/article/10.3390/antibiotics14101051/s1, Figure S1. Scatter plots showing the correlations between DASC/DOT and each indicator (DOT/100 prescriptions, DPM, and DPY) corresponding to Table 4.

## Abbreviations

AMR: Antimicrobial Resistance; DASC, Days of Antibiotic Spectrum Coverage; ASC, Antibiotic Spectrum Coverage; DOT, days of therapy; AUD, antimicrobial use density; DDDs, defined daily doses: DPM, DDDs/1000 prescriptions/month; DPY, DDDs/100 prescriptions/year.
