# Supplementary material for: Days of Antibiotic Spectrum Coverage (DASC) and Oral Antimicrobial-Use Trends at a Community Pharmacy in Japan: A 2018–2023 Retrospective Observational Study
Source: Antibiotics (Basel). 2025 Oct 21;14(10):1051. doi: 10.3390/antibiotics14101051 (PMC12561576; doi:10.3390/antibiotics14101051)
Supplement: Supplementary file 1 [file antibiotics-14-01051-s001.zip › antibiotics-3852438-supplementary.pdf]

Days of Antibiotic Spectrum Coverage (DASC) and Oral Antimicrobial-Use Trends at a Community Pharmacy in Japan: A 2018–2023 Retrospective Observational Study

Kosuke Hasegawa [1,2], Shoji Seyama [2], Tomoko Mori [1], Yuriko Matsumura [1] and Hidemasa Nakaminami [2,*]

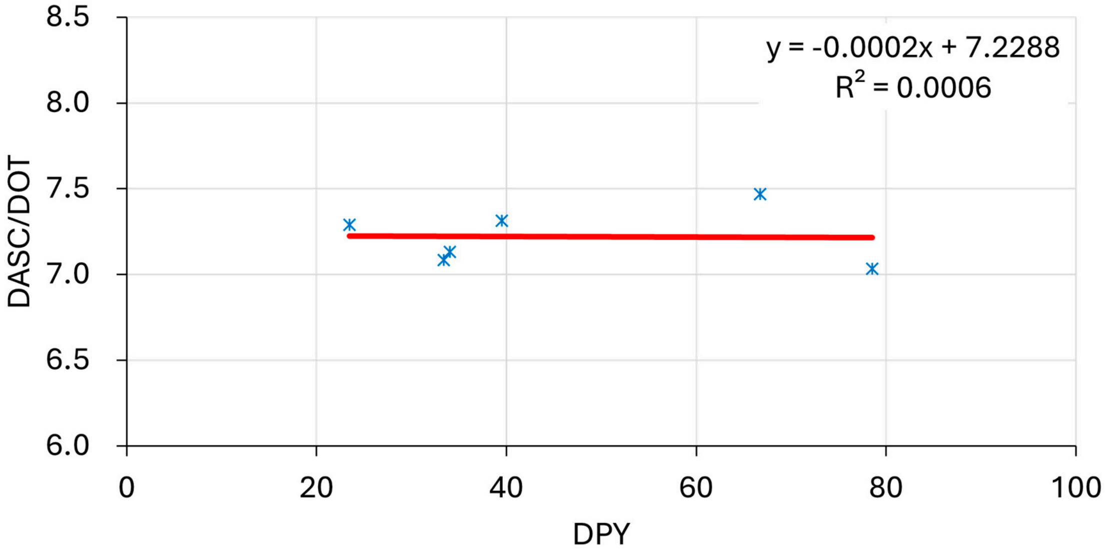

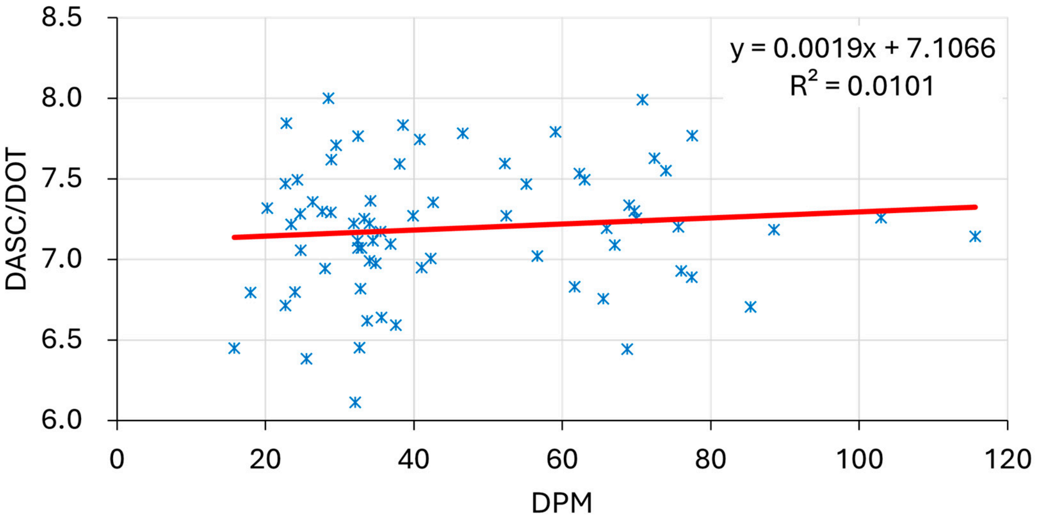

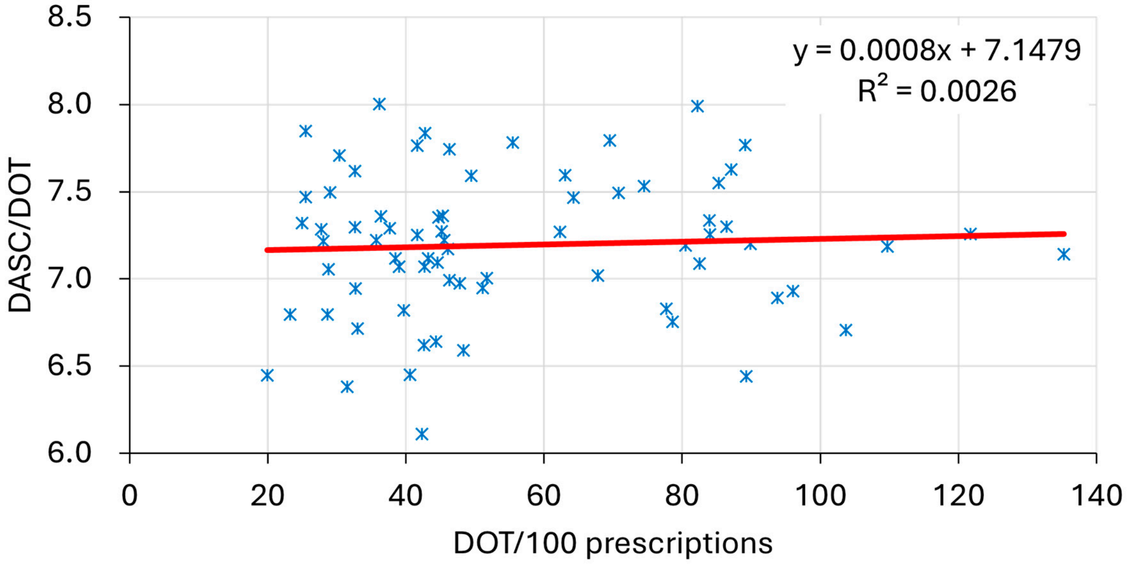

Figure S1. Relationship between monthly and annual antibiotic use and DASC/DOT.
